# Pharmacogenomics and Pharmacogenetics in Osteosarcoma: Translational Studies and Clinical Impact

**DOI:** 10.3390/ijms21134659

**Published:** 2020-06-30

**Authors:** Claudia Maria Hattinger, Maria Pia Patrizio, Silvia Luppi, Massimo Serra

**Affiliations:** IRCCS Istituto Ortopedico Rizzoli, Laboratory of Experimental Oncology, Pharmacogenomics and Pharmacogenetics Research Unit, 40136 Bologna, Italy; claudia.hattinger@ior.it (C.M.H.); mariapia.patrizio@ior.it (M.P.P.); silvia.luppi@ior.it (S.L.)

**Keywords:** osteosarcoma, pharmacogenomics, pharmacogenetics, toxicity, tailored treatment

## Abstract

High-grade osteosarcoma (HGOS) is a very aggressive bone tumor which primarily affects adolescents and young adults. Although not advanced as is the case for other cancers, pharmacogenetic and pharmacogenomic studies applied to HGOS have been providing hope for an improved understanding of the biology and the identification of genetic biomarkers, which may impact on clinical care management. Recent developments of pharmacogenetics and pharmacogenomics in HGOS are expected to: i) highlight genetic events that trigger oncogenesis or which may act as drivers of disease; ii) validate research models that best predict clinical behavior; and iii) indicate genetic biomarkers associated with clinical outcome (in terms of treatment response, survival probability and susceptibility to chemotherapy-related toxicities). The generated body of information may be translated to clinical settings, in order to improve both effectiveness and safety of conventional chemotherapy trials as well as to indicate new tailored treatment strategies. Here, we review and summarize the current scientific evidence for each of the aforementioned issues in view of possible clinical applications.

## 1. Introduction

Osteosarcoma, the most common tumor of the bone, is a very aggressive malignant neoplasm that mainly arises in adolescents and young adults [1,2,3]. Different variants of this neoplasm have been described, of which the most common is the conventional high-grade osteosarcoma (HGOS) that affects patients younger than 40 years, is localized in the extremities and does not present evidence of metastasis at clinical onset [2]. Conventional HGOS is currently treated with surgical removal of the primary tumor and systemic pre- and postoperative multidrug chemotherapy protocols. This multimodal, aggressive treatment allows curing 60–65% of patients, but also exhibits a collateral risk for adverse toxicity events that cannot presently be predicted or efficiently prevented [1,3,4,5,6]. The drugs that are most commonly included in first-line HGOS chemotherapy are doxorubicin (adriamycin), cisplatin (cis-diamminedichloroplatinum), methotrexate and/or ifosfamide and/or etoposide [3,5,7,8]. In addition to these, there is a small group of drugs, which, although they do not exhibit the same efficacy of the aforementioned ones, are variably used in second-line and rescue chemotherapy protocols to treat relapsed HGOS patients [3,4,7,9].

The ultimate goal of pharmacogenetics and pharmacogenomics (two terms that are frequently used interchangeably) is to define genomic variations, which may provide useful information to improve drug efficacy and reduce the risk of chemotherapy-related toxicities [10]. In the last decade, pharmacogenetic and pharmacogenomic approaches have been increasingly applied to the genetic characterization of HGOS and have started to yield insights which may be taken into consideration to modulate and, hopefully, improve the effectiveness and safety of currently used treatment protocols [5,11]. These studies are based on the application of genomic technologies to identify biomarkers related to drug efficacy or toxicity [12,13].

The majority of studies, which have been performed so far in HGOS, have evaluated single nucleotide polymorphisms (SNPs) in several candidate genes of possible relevance for the biology, drug response, detoxification and susceptibility to treatment-related toxicities, in order to indicate biomarkers which may provide the bases for planning tailored treatments aimed to increase therapeutic benefits and limit adverse events.

In this review, we provide an overview of the most promising translational research findings of pharmacogenetics and pharmacogenomics in HGOS. In particular, we have focused on both pharmacogenetic (germline) and pharmacogenomic (tumor-associated, somatic) markers, which have been indicated to influence treatment response, survival and susceptibility to treatment-associated toxicities in HGOS patients, thus appearing as promising candidates for a translation to clinical practice. 

## 2. Germline Pharmacogenetic Markers Associated with Risk to Develop HGOS 

Biological risk factors for HGOS include tall stature, high birth-weight and therapeutic irradiation [14]. Rare cancer predisposition syndromes have been described to be associated with HGOS. They are not a common cause of HGOS, but the incidence of HGOS is higher in these patients compared to the general population (Table 1). It cannot be excluded that by using next-generation sequencing (NGS) approaches additional genes associated with inherited syndromes will still be discovered, as was shown in the Diamond-Blackfan anemia (DBA) families [15,16].

The number of genes reported in the phenopedia database to be associated with HGOS has reached 170 [26] and will rise continuously. Although 117 genes have been reported in single studies and 35 in less than five, 18 genes have been reported more than ten times in association with osteosarcoma: excision repair cross-complementation group 1, 2 and 5 (*ERCC1, ERCC2, ERCC5*); X-ray repair cross complementing 3 (*XRCC3*); tumor protein 53 **(***TP53*); mouse double minute 2 (*MDM2*); cytotoxic T-lymphocyte antigen-4 (*CTLA-4*); glutathione S-transferase P1 (*GSTP1*); methylenetetrahydrofolate reductase (*MTHFR*); ATP-binding cassette, subfamily B (MDR/TAP) member 1 (*ABCB1*); ATP-binding cassette, subfamily C (CFTR/MRP) member 2 (*ABCC2*); ATP-binding cassette, subfamily C (CFTR/MRP) member 2 (*ABCC3*); glutathione S-transferase M1 (*GSTM1*); glutathione S-transferase T1 (*GSTT1*); cytochrome C oxidase subunit 8A (*COX8A*); vascular endothelial growth factor A (*VEGFA*); tumor necrosis factor (*TNF*); transforming growth factor beta 1 (*TGFB1*). Many of them belong to DNA repair pathways or drug metabolism, transport and detoxification of the principal drugs used in HGOS chemotherapy.

### 2.1. Common Genetic Variants

Common genetic variants have been studied mainly by candidate-gene and pathway driven approaches applied to smaller cohorts and have revealed somehow contradictory results. However, these studies indicated at least 27 genes to be associated with risk for HGOS development [14]. A very comprehensive candidate gene approach, evaluating 4836 tag-SNPs of 255 candidate genes belonging to four pathways with possible biological importance for HGOS risk in 96 HGOS cases and 1426 controls identified 241 SNPs that were statistically significant (*p* < 0.05) [27]. However, after correction for multiple testing, none of these pathways remained significantly associated with HGOS risk confirming the complex biology of HGOS pathogenesis.

The first international genome-wide association study (GWAS) using a high-throughput approach including 941 samples and 3291 controls identified SNPs in the glutamate metabotropic receptor 4 (*GRM4*) gene and in a gene desert at chromosome 2p25.2 which were found to be associated with risk to develop HGOS [28]. The risk association between *GRM4* rs11906953 and HGOS was subsequently confirmed in two studies carried out in Chinese populations [29,30].

A different approach was used by Yang and co-workers, who performed a gene-based GWAS analysis on a family-based trio data set including genotypes of 697,110 SNPs for 209 patients with HGOS and their unaffected biological parents [31]. Their objective was to identify height-related genetic markers associated with HGOS. They performed a Bayesian gene-based GWAS analysis in two steps. A total of 217 genes achieving genome-wide significance were identified. Ingenuity pathway analysis of this gene set indicated that genes were highly related to *TP53*, estrogen receptor signaling, xenobiotic metabolism signaling and RANK signaling in osteoclasts suggesting these pathways being associated with HGOS.

### 2.2. Rare Genetic Variants

Rare genetic variants have been studied in HGOS patients by targeted gene sequencing of *TP53* [32] and in three pan-cancer cohorts including HGOS using whole-exome (WES) or whole-genome (WGS) sequencing [33,34,35]. The first, performing WGS or WES on 1120 pediatric cancer patients (39 HGOS), identified *TP53, APC,* breast related cancer antigen 2 (*BRCA2*), neurofibromin 1 (*NF1*), PMS1 homolog 2, mismatch repair system component (*PMS2*), RB transcriptional corepressor 1 (*RB1*) and RUNX family transcription factor 1 (*RUNX1*) to be affected by mutations in more than three cases [35]. The second, reporting WES data on germline DNA samples from 1162 prevalently adult sarcoma patients (124 HGOS) found that 55% of patients had an excess of pathogenic germline variants in the 72 pan-sarcoma genes, such as *TP53, BRCA2,* ataxia telangiectasia mutated (*ATM*), ataxia telangiectasia and Rad3 related (*ATR*), and *ERCC2* [33]. The third, performed on 961 childhood cancer patients, suggested that 7–8% of the children carried an unambiguous predisposing germline variant [34]. 

Recently, the most comprehensive study reported WES and targeted sequencing data of germline DNA from 1244 patients with osteosarcoma in order to investigate the underlying germline genetic architecture [36]. Results were compared with 1062 in-house cancer free adult controls and 27,173 from the ExAC non-Finish European cancer-free resource. Analyses focused on 238 high interest cancer-susceptibility genes (114 cancer-predisposing genes, 14 genes associated with DBA, and 110 cancer-associated genes previously described or reported in the catalogue of somatic mutations in cancer (COSMIC) with germline effects) followed by testing for mutational burden across 736 additional candidate genes (140 genes of the HuGE Phenopedia and 596 genes somatically altered in pediatric bone cancers or recurrent in any pediatric cancer based on COSMIC and annotation of published osteosarcoma somatic data). Variants were grouped in pathogenic (P) and likely pathogenic (LP) variants as in previous studies [32]. A significantly higher P/LP variant burden in the 238 high-interest cancer-susceptibility genes was found in the HGOS cases compared to the in-house controls restricted to European ancestry. A P/LP variant was identified in 28% of the cases, of which nearly three-quarters mapped to an autosomal dominant gene or a known osteosarcoma-associated cancer predisposition syndrome gene. Higher than expected frequencies of P/LP variants were found in genes previously linked to osteosarcoma, e.g., cyclin dependent kinase inhibitor 2A (*CDKN2A*), menin 1 (*MEN1*), Von Hippel-Lindau (*VHL*) tumor suppressor, protection of telomeres 1 (*POT1*), *APC*, mutS homolog 2 (*MSH2*), ATRX chromatin remodeler (*ATRX*) and *TP53*. 

### 2.3. Meta-Analyses

Several recent meta-analyses have been performed, including selected case-control studies, in order to obtain more robust results and to clarify the somehow contradictory results of individual small candidate gene and pathway driven studies. 

The *TP53* rs1042522 was studied in a meta-analysis including five publications with a total of 567 cases with bone tumors (HGOS and Ewing sarcoma) and 935 controls [37]. In the stratified group of patients with HGOS (527 patients and 807 controls) the GG genotype was significantly associated with risk for HGOS development vs GC/CC (odd ratio, OR = 1.57, confidence interval, CI = 1.20–2.06, *p* = 0.001). 

Polymorphisms of the GST family (*GSTP1* rs1695, *GSTT1* and *GSTM1* null allele, and *GSTM3* rs1799735 were analyzed in a meta-analysis including 24 case-control studies with a total of 2405 HGOS cases and 3293 controls [38]. All five genetic models were tested. Including all studies, the *GSTT1* null genotype (OR = 1.2, 95% CI 1.02–1.52, *P* = 0.031) and the *GSTP1* (rs1695) variant allele (B vs A: OR = 8.90, 95% CI 2.72–29.20, *p* < 0.001) were associated with higher risk for HGOS, whereas increased risk was also associated with the *GSTT1* null genotype (OR = 1.30, 95% CI 1.03–1.64, *p* = 0.025) but only in Asians and not in Caucasians.

The two most frequently studied variants of *MDM2*, rs1690916 and rs2279744, for which inconclusive data were reported regarding a possible association with risk to develop HGOS [39,40,41] were considered in a meta-analysis including six populations with a total of 246 HGOS patients and 1760 controls (rs2279744) and 433 HGOS patients and 1959 controls (rs2279744) [42]. None of the two *MDM2* SNPs was associated with risk to develop HGOS in any of the four models studied, suggesting that the role of MDM2 in the development of HGOS is less important than was previously expected.

A more comprehensive approach was used in a one meta-analysis considering 32 case-control studies with a total of 6924 HGOS patients and 8412 controls, and 24 SNPs in 14 genes [43]. Nine risk alleles (*CTLA-4* rs231775; protein kinase CGMP-dependent 1, *PRCKG* rs454006; RecQ like helicase 5, *RECQL5* rs820196; *TNF-a* rs1800629; *TP53* rs1042522; *XRCC3* rs861539; *VEGF* rs699947 and *VEGF* rs3025039) with an average pooled OR = 2.082 (CI 1.59–3.26; *p* < 0.003) and three protective alleles (interleukin 8, *IL-8* rs4073; *MDM2* rs1690916; and *VEGF* rs2010963) with OR = 0.61 (0.51–0.72, *p* < 0.038) were identified. However, the authors admit that one of the limitations of their study was that most of the studies included Chinese populations and therefore their data should be validated in different ethnic populations.

## 3. Pharmacogenetic and Pharmacogenomic Markers Associated with Treatment Response and/or Survival

**Figure 1:**
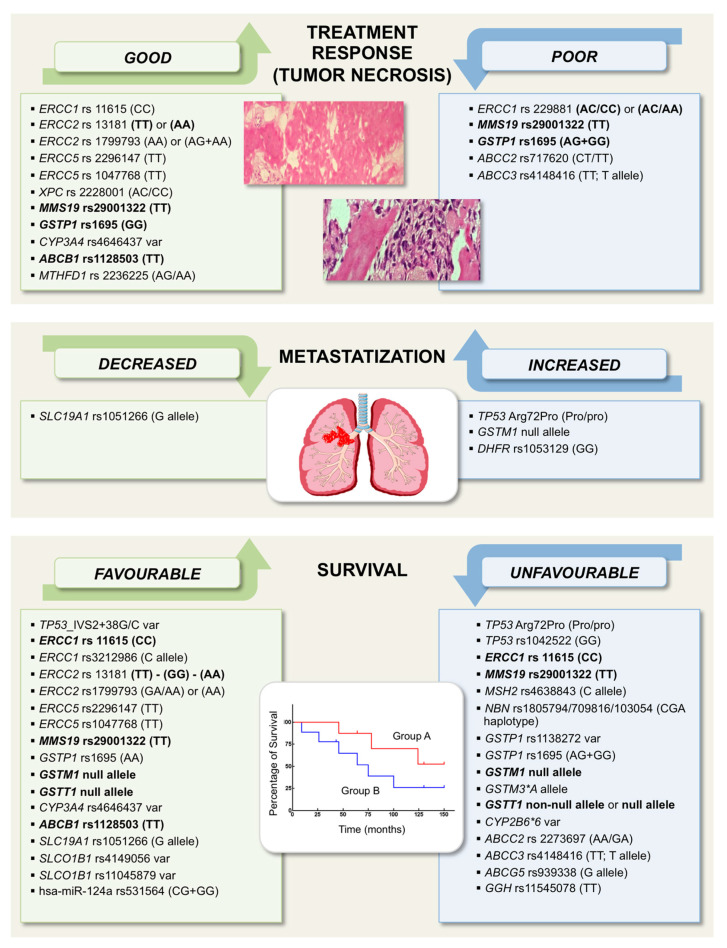
Pharmacogenetic and pharmacogenomic variations impacting on treatment response, outcome and prognosis in high-grade osteosarcoma (HGOS). Genes, polymorphisms and corresponding genotypes (inside parenthesis) are listed inside each box. Genotypes have been reported as they were described in the original studies. Gene polymorphisms or genotypes for which contradictory results have been reported are marked in bold. Genes polymorphisms included in the green boxes (left side) are those which have been indicated to favorably impact on patients’ outcome. Further details, abbreviations legend and references are provided in the text.

Studies on genetic markers that may be associated with treatment response and outcome in HGOS have mainly focused on genes involved in tumorigenesis, DNA repair pathways, as well as in metabolism, transport, or detoxification of drugs used for conventional chemotherapy protocols. The most relevant studies and candidate biomarkers with a possible clinical impact which have been reported so far are summarized below and listed in Figure 1. 

### 3.1. TP53 and Its Regulators MDM2 and Mouse Double Minute 4 (MDM4)

The significance of mutations and genetic abnormalities of the *TP53* gene for bone and soft tissue sarcoma pathogenesis and progression has been supported by several studies, which have proven the high frequency and variability of *TP53* mutations in HGOS [44,45]. However, recent WGS analyses have indicated that the significance of *TP53* alterations in bone and soft tissue sarcomas could have been underestimated [46]. This fact has strengthened the interest for *TP53* and its pathway as possible therapeutic targets, especially for tumors that, like HGOS, frequently present alterations of this gene [46].

The *MDM2* gene, a crucial regulator of the TP53 protein stability and degradation, has been found to be amplified and overexpressed in a relevant number of HGOS [39,47].

Another major inhibitor of TP53 is *MDM4*, which encodes for a protein that, such as MDM2, binds to TP53, inhibiting its activity [48]. *MDM4* amplification has been revealed in 35% HGOS [39,49] and it has been associated to poor prognosis in HGOS, soft tissue sarcomas, and other cancers [50,51,52]. 

Inhibitors of MDM4, TP53-MDM2 interaction, or MDM2/MDM4 dual inhibitors have been tested in clinical trials or are presently under investigation [46], but their efficacy in HGOS must still be determined. While current TP53-targeted therapies have shown severe limitations in clinical settings, mostly due to induction of bone marrow suppression and other adverse side effects, they keep a great interest as candidate approaches that might be used to treat therapy-resistant bone and soft tissue sarcoma patients in the near future. However, obtaining details on tumor genotype of patients who may be potentially eligible for these treatments is mandatory prior starting any targeted therapy.

Although *TP53* and *MDM2* SNPs have been extensively reported as risk factors for HGOS [31,39,41,43,44,53,54,55], much fewer information is available about their impact on clinical outcomes of HGOS patients. 

The *TP53* rs1800372 (p.R213R), a rare synonymous variant leading to an exonic splice site change, was described to be associated with higher incidence of metastasis at diagnosis in European HGOS patients [32].

The *TP53* rs1042522 Arg72Pro polymorphism (Pro/Pro *versus* wild-type Arg/Arg genotype) was reported to be associated with increased risk of relapse and to have an adverse prognostic value for event-free and overall survival in HGOS patients [40]. 

In a series of 210 HGOS Chinese patients, the GG genotype of the *TP53* rs1042522 polymorphism was found to be associated with a shorter survival time compared with patients carrying the CC genotype [56].

The variant status of *TP53*_IVS2+38G/C rs1642785 polymorphism was reported to be associated with better event-free survival in a group of 126 HGOS [54]. 

Concerning *MDM2*, a meta-analysis indicated that polymorphisms affecting this gene are related to HGOS risk, but do not seem to have effects on patients’ survival, even if further studies are needed to confirm the last assumption [41].

### 3.2. DNA Repair-Related Genes

The altered activity of factors involved in the repair of drug-induced DNA damages can significantly influence either the resistance or sensitivity to DNA targeting drugs, including those used in HGOS chemotherapy [57,58]. Consequently, it is not surprising that polymorphisms of genes belonging to DNA repair pathways have proved to be associated with drug response, survival and toxicity in different tumors [57,59]. 

The available scientific literature has shown that several polymorphisms affecting DNA repair genes appear to variably correlate with treatment response and/or survival also in HGOS, but the reported findings were sometimes contradictory, as summarized below. 

The CC genotype of the *ERCC1* rs11615 polymorphism was found to be associated with good response to cisplatin-based chemotherapy and better overall survival when compared to TT genotype [60,61,62,63,64]. However, other studies described the opposite evidence, being a better survival associated with the TT compared to CC genotype [65,66].

Patients carrying the C allele of the *ERCC1* rs3212986 polymorphism were described to have better event-free survival [67].

Association with poor response to chemotherapy was reported for the AC/CC genotype of the *ERCC1* rs2298881 polymorphism [62], but another study provided partially opposite findings indicating that the AC/AA genotype associated with poor chemotherapy responsiveness [60]. 

Concerning the *ERCC2* rs13181 polymorphism, the TT genotype was found to be associated with better treatment response and event-free survival compared to GT/GG genotype [68], but there were also studies reporting the correlation with a better event-free survival for the GG compared to TT genotype [69] or for the GG compared to AA genotype [70]. The AA genotype was also described to be associated with higher treatment response and increased overall survival compared with GG genotype [71]. 

An association with better event-free survival was reported for the GA/AA genotypes of *ERCC2* rs1799793 polymorphism compared to GG genotype [69] and for patients with at least one polymorphic allele (GA/AA) [72]. In concordance with this evidence, other two studies showed that the AA genotype correlated with better response to chemotherapy and overall survival compared with the GG genotype [66,73].

Better response to cisplatin-based chemotherapy and overall survival was described for the TT genotype of the *ERCC5* rs2296147 and rs1047768 polymorphisms [74,75,76]. 

The TT genotype and T allele of the nucleotide excision repair homolog (*MMS19*) rs29001322 polymorphism resulted to be associated with better response to cisplatin-based chemotherapy and increased overall survival [74,76]. However, the opposite evidence for TT genotype in relation to survival was reported in another study [75].

An association with worse outcome was found for patients carrying the C allele of the *MSH2* rs4638843 polymorphism [77].

The nibrin (*NBN*) rs1805794+rs709816+rs1063054 CGA haplotype was found to be associated with shorter event-free survival compared with CAA haplotype [72].

Patients carrying the AC/CC genotypes of xeroderma pigmentosum complementation group C (*XPC*) rs2228001 polymorphism showed higher response rates to pre-operative chemotherapy [68]. 

Several studies performed on HGOS have recruited limited numbers of patients, as it is frequently the case for rare tumors. In the attempt to overcome, at least in part, this limitation, meta-analyses have been performed with the aim to support the translation of provided evidence into clinical practice. 

In particular, three meta-analyses focused on the most frequently studied polymorphisms of nucleotide excision repair (NER) genes in HGOS [63,70,78].

Two of these studies confirmed that HGOS patients carrying the C allele of *ERCC1* rs11615 polymorphism showed better response to chemotherapy in both Caucasian and Chinese populations [63,78]. In Chinese HGOS patients, a higher chemotherapy response rate was also found for *ERCC2* rs1799793 (AG + AA) genotypes [63]. The third meta-analysis indicated that the AA genotype of *ERCC2* rs13181 polymorphism was associated with a better survival rate compared to the GG genotype in both Chinese and Caucasian HGOS patients [70].

In general, despite the additional information provided by meta-analyses, it can be concluded that the findings reported so far concerning DNA repair-related gene polymorphisms in HGOS must be further confirmed, in order to identify the possible reasons for discrepancy and, subsequently, to validate their predictive and prognostic value before being considered for a real clinical translation.

### 3.3. Genes Involved in Drug Metabolism

Chemotherapeutic agents are detoxified and, in the case of prodrugs, activated by several drug metabolizing enzymes (DMEs), which are responsible for their biotransformation in both tumor and normal cells [79]. It is therefore easily understandable that DMEs can significantly influence the tumor responsiveness to chemotherapy, as well as the susceptibility of normal tissues to treatment-related toxicities. Pharmacogenetic and pharmacogenomic analyses of DMEs in tumor patients may thus provide useful indications to modulate the use of chemotherapeutic drugs according to patients’ genetic characteristics.

When considering the possible clinical impact of DMEs, the role of each enzyme for its specific drug biotransformation must be taken into account. For example, gene variations resulting in a DME function impairment can decrease the therapeutic activity of pharmacologically inactive prodrugs, which require bioactivation to become effective, but also be associated with a lower induction of toxicity in normal cells. On the other hand, regarding DMEs involved in drug detoxification, gene alterations associated with an impaired enzyme activity can determine an increased therapeutic effectiveness, but also a higher risk for collateral toxicity. 

In the last 15 years, the number of genetic studies concerning DMEs in HGOS has been progressively increased and has provided some interesting information. 

Glutathione S-transferase (GST) enzymes play a major role in the detoxification of a wide range of chemotherapeutic drugs [80] and are the DMEs that have most widely been studied in HGOS. Different polymorphisms of the *GSTP1* gene have been reported to be associated with a variable enzyme ability to metabolize anticancer agents [81] and, for some of them, evidence of a possible clinical impact in HGOS has been reported, even if findings were sometimes contradictory or, at least, not completely concordant. This is the case for studies that analyzed the *GSTP1* rs1695 polymorphism, which have provided discordant indications. The *GSTP1* rs1695 AG + GG genotypes have mostly been reported to be associated with poor histological response and worse survival in HGOS patients treated with neo-adjuvant chemotherapy [82,83,84,85,86], but a large Chinese study showed that the GG genotype was related to good response to chemotherapy [71]. 

The most relevant, but again not always concordant, results concerning the other GST isoenzymes can be summarized as follows:

- The *GSTM1* null allele was reported to be associated with an increased relapse rate in non-metastatic patients, and with poor survival in metastatic patients [87].
- The non-null allele of *GSTT1* was correlated with poor survival in metastatic patients [87]. On the other hand, poor event-free survival was shown to be associated with the *GSTT1* null allele in a study on Caucasian patients [85]. Partially in contrast with these findings, *GSTM1* and/or *GSTT1* null genotypes were reported to be associated with better survival rates in a Chinese study [86].
- The GSTM3*B allele of rs1799735 was reported to be associated with the presence of metastases at diagnosis, whereas the GSTM3*A allele rs1799735 emerged to be correlated with worse survival in patients presenting metastasis at clinical onset [87,88].

The aforementioned body of evidence has been generated from analyses on germline pharmacogenetic variants, but the clinical impact of GSTs pharmacogenomic, somatic variants has also been assessed. In a study performed on tumor tissue from 66 HGOS patients, the *GSTP1* rs1138272 variant genotype proved to be associated with both shorter event-free and overall survival, whereas no association with survival was revealed for deletions of either *GSTM1* or *GSTT1* [72]. 

Meta-analyses about GST polymorphisms in HGOS provided apparently contradictory results. One of these studies [89] revealed significant associations between the AA genotype of *GSTP1* rs1695 polymorphism and good tumor response, progression-free and overall survival. Another meta-analysis, however, did not find significant associations with clinical parameters of polymorphisms affecting GSTP1, GSTM3, and GSTT1 [90]. A third meta-analysis performed on 681 Chinese Han patients, did not find any association between GSTM1 and GSTT1 polymorphisms and chemosensitivity [91].

Other very important DMEs are those belonging to the cytochrome P450 (CYP) family. CYPs are involved in both anticancer drugs detoxification and prodrugs activation, therefore playing a key role in determining drug effectiveness and collateral toxicity [92]. Since these enzymes show a relevant genetic variability, CYPs polymorphic variants can lead to differences in treatment response and susceptibility to chemotherapy-associated toxicities.

The reported evidence with the most relevant clinical impact in HGOS concerns the *CYP3A4* rs4646437 polymorphism [77]. The variant allele of *CYP3A4* rs4646437 was shown to be associated with better treatment response and survival in HGOS patients treated with standard neoadjuvant chemotherapy protocols, most probably due to the reduced CYP3A4-mediated drug inactivation [77].

The variant genotype of the CYP2B6*6 (rs3745274 and rs2279343) polymorphism was found to be associated with a worse event-free survival, probably because of a decreased enzyme expression and a consequent decreased ifosfamide activation [54].

As a general conclusion, it can be stated that, despite this very promising body of results, stronger evidence is needed to definitely prove the clinical impact of DMEs genetic alterations in HGOS pathogenesis and prognosis.

### 3.4. Genes Involved in Drug Transport

Drug traffic into and out of cells is mediated by specific membrane transporters, which play a key role in determining tissue drug concentrations and, consequently, their therapeutic efficacy and toxicity on normal tissues of anticancer agents. Concerning HGOS, evidence of correlation with clinical parameters has been reported for transporters belonging to the ATP binding cassette (ABC) family. Several first- and second-line HGOS chemotherapeutic drugs are substrates of these transporters and, therefore, it is not surprising that polymorphisms affecting members of this family have been indicated as candidate biomarkers for a possible clinical translation. Gene polymorphisms that have reported to be significantly associated with therapy response and /or survival in HGOS can be summarized as follows:

- The TT genotype of the *ABCB1* rs1128503 polymorphism was related to good chemotherapy response and better survival in three studies [83,93,94], but some discrepant observations were reported in other two analyses [82,95];
- The CT/TT genotypes of the *ABCC2* rs717620 polymorphism were shown to be associated with a poor response to pre-operative chemotherapy [85], whereas the AA/GA genotypes of the *ABCC2* rs2273697 polymorphism correlated with worse event-free survival [54];
- The variant status of the *ABCC2*_1249A/G (rs2273697) polymorphism was reported to be associated with worse event-free survival [54];
- Association with poor response to first-line chemotherapy and worse survival was described for the TT genotype and T allele of the *ABCC3* rs4148416 polymorphism [83,93,95];
- Worse survival was found to be associated with the G allele of the *ABCC5* rs939338 polymorphism [77].

A meta-analysis confirmed the association of *ABCB1* rs1128503 (TT genotype) with good response and of *ABCC3* rs418416 (T allele) with poor response to chemotherapy in Caucasian populations [96]. 

In summary, again, if promising findings for a possible clinical translation have been reported for ABC transporters, further validation is warranted to validate the reliability of these candidate genetic markers.

### 3.5. Polymorphisms of Genes Involved in Antifolate Drugs Metabolism

One of the most widely used drug for HGOS treatment is antifolate methotrexate (MTX). Two other antifolates, trimetrexate and pemetrexed, have been used in second-line chemotherapy protocols to treat recurrent HGOS patients, but their activity proved to be unfortunately modest [97,98,99]. 

Pharmacogenetic and pharmacogenomic studies in HGOS have focused on the analysis of polymorphisms affecting genes that play important roles in the metabolism of MTX and other antifolate drugs. The data with evidence of clinical correlations in HGOS are summarized below.

The GG genotype of the rs1053129 polymorphism of the dihydrofolate reductase (*DHFR*) gene, the main target of MTX, was reported to be associated with a higher probability to develop metastasis during follow-up [85].

The rs1051266 polymorphism of the solute carrier family 19 folate transporter member 1 (*SLC19A1*) gene (also known as reduced folate carrier, *RFC* or reduced folate carrier 1, *RFC1*), a MTX membrane transporter, was reported to be associated with clinical outcome, showing that patients bearing the allele G had better survival and lower predisposition to develop metastases [85,100].

The AG/AA genotype of the rs2236225 polymorphism of the methylenetetrahydrofolate dehydrogenase (NADP+ dependent) 1 (*MTHFD1*) gene, a folate cycle enzyme which is involved in de novo purine synthesis, was found to be associated with good histological response after preoperative chemotherapy [85].

The TT genotype of the rs11545078 polymorphism of the gamma-glutamyl hydrolase (*GGH*) gene, an enzyme which catalyzes the hydrolysis of folylpoly-gamma-glutamates and anti-folylpoly-gamma-glutamates influencing the overall effectiveness of MTX, was found to be associated with poor survival [54].

Another gene that has been studied in HGOS is the solute carrier organic anion transporter family member 1B1 (*SLCO1B1*), which is the main uptake transporter of MTX in the liver and one of the most important factors influencing MTX clearance. Patients with at least one polymorphic allele of *SLCO1B1* rs4149056 and rs11045879 polymorphisms exhibited longer event-free survival compared to patients with two wild-type alleles [101]. 

By considering the studies reported so far in HGOS concerning polymorphisms affecting genes involved in the MTX and folate pathways, it must be underlined that the numbers of patients carrying genotypes associated with unfavorable treatment response or prognosis were invariably low, and, therefore, all this body of evidence needs further validation in larger patients series before an effective translation into clinical practice. 

## 4. Gene Polymorphisms Associated with Toxicities

High-dose methotrexate often causes bone marrow suppression and liver and renal toxicities, whereas anthracyclin-induced cardiotoxicity and cisplatin-induced ototoxicity have been reported [102]. Mainly candidate gene and pathway driven approaches have revealed some recurrent associations between gene variations and collateral toxicities in HGOS patients [103]. Confirmation of this evidence in a larger patient series is needed before this knowledge can be translated into personalized treatment schedules, except for cisplatin-induced ototoxicity and doxorubicin-induced cardiotoxicity for which international recommendations have already been provided [104,105]. Since the possible functional role of most of these genetic variants is not yet clear and can be different in normal and tumor cells, further functional studies are needed to elucidate their biological consequences in association with cell death, drug detoxification and transport in both normal and tumor tissue. 

### 4.1. Haematological Toxicities

**Figure 2:**
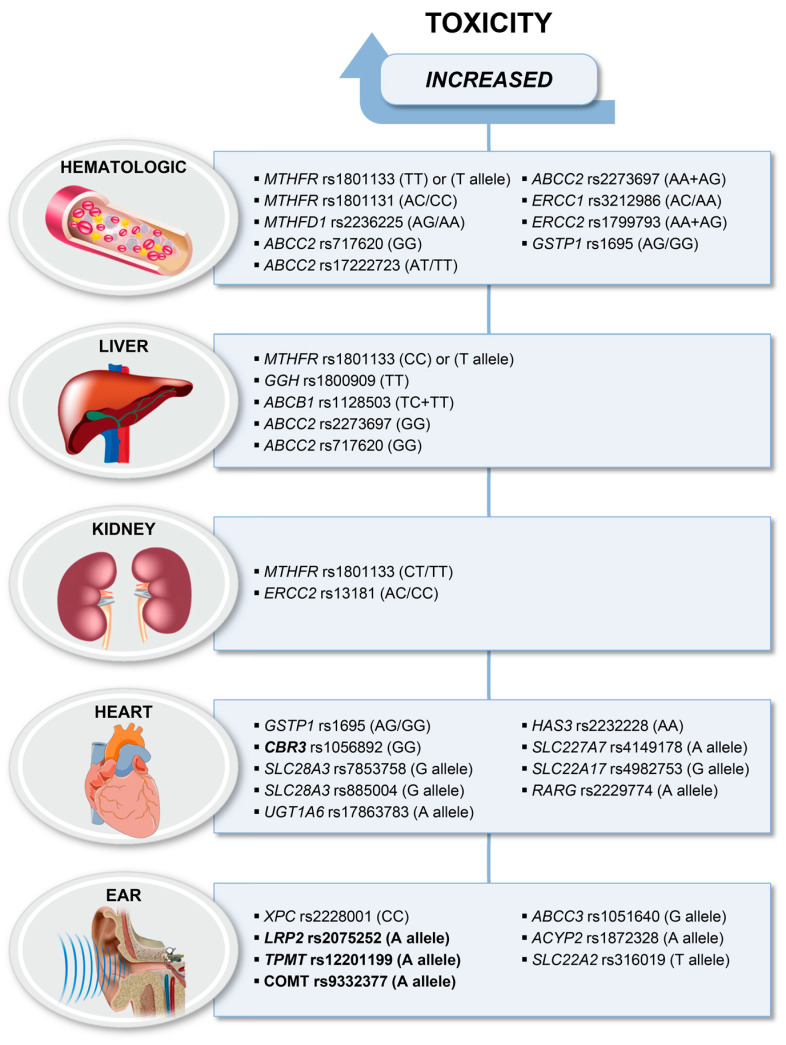
Pharmacogenetic variations impacting on chemotherapy-induced toxicities in high-grade osteosarcoma (HGOS). Genes, polymorphisms and corresponding genotypes (inside parenthesis) are listed inside each box. Genotypes have been reported as they were described in the original studies. Genes polymorphisms or genotypes for which contradictory results have been reported are marked in bold. Further details, abbreviations legend and references are provided in the text.

As shown in Figure 2, genes reported in association with hematological toxicity belong to the folate or NER pathways, the ABC transporter family or to DMEs. Pharmacogenomics of genes belonging to the folate metabolism or transport in HGOS patients have recently been reviewed [106]. 

The 5,10-methylene tetrahydrofolate reductase (*MTHFR*) rs1801133 polymorphism leads to a C to T substitution at nucleotide position 677, with the result of a substitution of valine for alanine in the functional enzyme and a decreased enzymatic activity. The TT genotype was significantly associated with grade 3–4 hematologic toxicity in 96 pediatric HGOS patients [107]. In a recent study, the variant T allele of this polymorphism was reported to correlate with higher degrees of hematologic toxicities in 59 Han Chinese patients with HGOS [108]. 

A second polymorphism of the *MTHFR* gene, rs1801131, leads to an A to C substitution at nucleotide 1289 and also to reduced enzyme activity. The AC/CC genotypes were found to be associated with anemia and with severe leukopenia in 50 [85] and 57 HGOS patients [54]. 

AG/GG genotypes of a third gene polymorphism belonging to the folate pathway, the *MTHFD1* rs2236225, were reported to be associated with anemia [85]. 

Among the ABC transporter gene family, three polymorphisms of the *ABCC2* gene, rs717620 (GG) [101], rs17222723 (AT/TT) [85] and rs 2273697 (AA + GA) [54,109] have been reported to be associated with hematological toxicities. 

The AC/AA genotypes of *ERCC1* rs3212986 and the AG/GG genotypes of *GSTP1* rs1695 were reported to be associated with leukopenia in one study [85]. Another polymorphism of the NER pathway, the AA/AG genotype of *ERCC2* rs1799793, was reported in association with thrombocytopenia [54].

### 4.2. Liver Toxicity

The *MTHFR* rs1801133 polymorphism has been reported by different groups to be associated with liver toxicity [110]. One study carried out in patients after high-dose MTX-therapy found a significant association between the T allele and liver toxicity in 49 patients with HGOS [111]. This association, however, was not confirmed in the subsequent meta-analysis, including seven studies with a total of 1044 patients (148 with HGOS), whereas a recent study on 59 Han Chinese patients with HGOS found the variant T allele of *MTHFR* rs1801133 being associated with higher degrees of liver toxicity [108]. 

Apparently contradictory results were reported in a Norwegian study on 62 patients with HGOS [100]. The CC genotype of *MTHFR* rs1801133 was associated with a higher degree of liver toxicity. Although statistically not significant, patients who could not receive the whole number of MTX cycles because of collateral toxicity were more frequently carriers of the CC and AC genotypes. 

A very small descriptive study of 18 patients, including seven with HGOS who had developed severe clinical or liver toxicity measured by elevated alanine transaminase (ALT) levels revealed that rare alleles of *MTHFR* rs1801133 and thymidylate synthase (*TS*) rs34743033 were more frequent in HGOS patients compared to the normal population [112].

The TT genotype of *GGH* rs189909, another gene of the folate pathway, was reported in association with hepatotoxicity in one study [54].

Among the ABC transporter genes, two polymorphisms of the *ABCC2* gene, rs717620 (GG) [101] and rs2273697 (GG), and *ABCB1* rs1128503 (TC + TT) have been reported to be associated with liver toxicity [54]. 

### 4.3. Nephrotoxicity

Although renal failure is frequent when high-dose MTX and cisplatin are administered, only few studies could find associations with genetic polymorphisms. Mueller and co-workers [113] described one patient who was homozygous for the variant of *MTHFR* rs1801133 (TT) but wild-type for *MTHFR* rs1801131 (AA). They concluded that the association between *MTHFR* rs1801133 TT and severe MTX-related toxicity could be explained by disturbances in the folate status and by prolonged MTX exposure due to delayed MTX clearance. 

One study reported that patients with the variant genotypes GA/AA of *SLC19A1* rs1051266 had significantly lower MTX plasma levels at 48 and 72 h, whereas the GG genotype was associated with higher plasma levels and nephrotoxicity [114]. 

Similar evidence was reported in 50 HGOS patients carrying the variant allele of *MTHFR* rs1801133 (CT/TT) and of *ERCC2* rs13181 (AC/CC) [85]. For both polymorphisms, a trend for association with early nephrotoxicity was found.

### 4.4. Cardiotoxicity

Pharmacogenetic studies of anthracycline-induced cardiotoxicity (ACT) in patients with HGOS have recently been reviewed [103]. First, evidence was provided by a candidate gene study in 50 patients with HGOS that the *GSTP1* rs1695 variant allele was associated with cardiotoxicity [85], but this result was not confirmed by others. 

Several large studies on pediatric cancer patients including HGOS have focused on drug metabolism genes with possible impact on cardiotoxicity (Figure 2). Carbonyl reductases (CBRs) are involved in the reduction of anthracyclines. The variant GG genotype of the *CBR3* rs1056892, which influences the synthesis of these metabolites, was associated with cardiomyopathy in patients who received low-to-moderate-dose but not in patients who received high-dose anthracyclines [115]. The authors concluded that there was no safe dose for patients homozygous for the variant G allele. However, this association was not confirmed in a large international study using a customized assay containing almost 3000 SNPs of 220 key biotransformation genes [116]. They identified a strong association between the variant G allele of solute carrier family 28 member 3 (*SLC28A3*) rs7853758 and ACT which was confirmed in a subsequent study by the same authors [117]. In addition, associations between ACT and a second variant of *SLC28A3*, rs885004, and the variant rs17863783 in UDP glucuronosyltransferase 1 A6 (*UGT1A6*) were confirmed in this replication study including 16 patients with HGOS of the total cohort of 202 pediatric cancer patients. In another large study including pediatric patients with bone tumors, using a cardiovascular SNP array to profile common SNPs in 2100 genes considered relevant to de novo cardiovascular disease, the AA genotype of rs2232228 in the hyaluronan synthase 3 (*HAS3*) gene conferred an increased risk for cardiomyopathy compared to the AA genotype [118]. This study did not include the *CBR3* and *SLC28A3* variants of the previous studies. 

In order to identify new variants and to improve their previously reported genotype-guided risk prediction model, Visscher and co-workers genotyped two cohorts of pediatric cancer patients for 4578 SNPs in absorption, distribution, metabolism, excretion (ADME) and toxicity genes [119]. Two novel SNPs, rs4982753 in solute carrier family 22 member 17 (*SLC22A17*) and rs4149178 in solute carrier family 22 member 7 (*SLC22A7*), were identified to be significantly associated with ACT. The same group performed a GWAS study in which they identified the new non-synonymous variant rs2229774 in the retinoic acid receptor γ (*RARG*) gene to be associated with ACT in childhood cancer [120]. All this evidence has formed the basis for the recommendations published by the same authors to guide genetic testing in pediatric patients in order to avoid ACT (PharmGKB ID PA166159180). They state that *RARG* rs2229774, *SLC28A3* rs7853758 and *UGT1A6* rs17863783 variants currently have the strongest and the most consistent evidence for association with ACT.

A recent study carried out on 167 anthracycline-exposed childhood cancer survivors (75 cases and 92 matched controls with different diagnosis including 40 with bone tumors) reported that patients who had the GSTM1 null genotype had a significantly higher risk to develop cardiomyopathy [121]. GSTM1 expression downregulation in patients with the null phenotype in peripheral blood was confirmed by array and RNA-Seq analyses. In addition, gene expression analysis of human-induced pluripotent stem cell cardiomyocytes from patients who had ACT showed significantly reduced expression of GSTM1 compared to those from patients without ACT. The authors conclude that also GSTM1 could be considered in a risk-prediction model to facilitate the identification of childhood cancer survivors who are at increased risk of anthracycline-related cardiomyopathy.

### 4.5. Ototoxicity

Pharmacogenetic studies reporting data regarding cisplatin-induced ototoxicity in patients with HGOS were mainly candidate gene-driven focusing on genes expressed in different tissues of the ear, involved in drug detoxification or on DNA repair genes [103]. Since many of them reported protective effect of gene variants which were not verified in replicate studies, Figure 2 shows only those polymorphisms that were found to be significantly associated with ototoxicity. 

In a subgroup of 32 HGOS patients, the CC genotype of *XPC* rs2228001 was associated with ototoxicity [68]. The variant allele A of the LDL receptor related protein 2 (*LRP2*) rs2075252 was reported in association with ototoxicity in a series of 38 patients with HGOS [122], but no evidence of this association was found in the study by Ross and co-workers [123]. Another candidate-gene study reported that the variant T allele of the solute carrier family 22 member 2 (*SLC22A2*) rs316019 was associated with hearing loss in 41 patients with HGOS [124]. 

Variants of thiopurine S-methyltransferase *TPMT* (rs12201199, rs180046 and rs1142345) and catechol O-methyltransferase (COMT) (rs4646316 and rs9332377) were reported to be associated with ototoxicity after treatment with cisplatin in different cancers, including HGOS [123,125]. In the only study of exclusively patients with HGOS (*n* = 139), no associations between these variants and increased risk of ototoxicity were reported. However, the meta-analysis including a total of 664 patients revealed that the *COMT* rs4646361 was the only one that remained significant but associated with less risk (OR = 1.52) as predicted in the other studies (OR = 2.52) [126]. Although initially promising, variants in the *COMT* and *TPMT* genes do not seem to play major roles as risk factors to develop cisplatin-induced ototoxicity and therefore stronger markers for clinical decision making are warranted. However, pharmacogenetic testing for the *TPMT* alleles *2 (rs1800462), *3A (rs1800460 and rs1142345), *3B (rs1800460) or *3C (rs1142345) is recommended by the Canadian Pharmacogenomics Network for Drug Safety in pediatric cancer patients when prescribing cisplatin (PharmGKB ID PA166170751).

The acylphosphatase 2 (*ACYP2*) rs1872328 was reported first in children with brain tumors [127] and was then confirmed in patients with HGOS [128].

Protective effects were reported for the GSTM3* rs1799735 allele in a group with normal hearing [129] and for the G alleles of *Otos* rs77124181 (c.-192-182C>G) and rs2291767 (c.-192-22A>G) [130]. 

### 4.6. Other Treatment-Related Toxicities

In addition to the toxicities that can result also in the long-term insufficiencies described above, several polymorphisms have been reported in association with less severe but frequent toxicities influencing the quality of life during chemotherapy treatment. In a study on tumor tissue obtained from 66 HGOS patients, the CC genotype of *XRCC3* rs 861539 was associated with nausea [72]. The same study reported the GA/AA genotype of *ERCC2* rs1799793 to be associated with nausea and gastrointestinal toxicity [72]. Similar evidence was reported for the CC/CT genotypes of *XRCC3* rs861539 being associated with nausea and vomiting, as was reported also for the GG genotype of *ABCC2* rs3740066 [54].

The following polymorphisms were reported in association with mucositis: the T allele of *DHFR* rs1650723 and the CT genotype of *ABCG2* rs2231135 [100], the TT genotype of *ERCC1* rs1615 and CT/CC genotypes of *ABCB1* rs1045642 after MTX treatment [85], and the GG genotype of *SLC19A1* rs1051266 [114].

Two studies reported polymorphisms in association with fever: the AC/AA genotypes of *ERCC1* rs3212986 [54] and the T allele of *MTHFR* rs1801133 [108].

## 5. Polymorphisms of Non-Coding RNAs

Non-coding sequences represent up to 98% of the human genome and, like coding DNA, can be affected by genetic changes [131]. Since the majority of non-coding elements can influence gene expression, genetic variants of non-coding regions may become relevant for clinical care through the altered modulation of gene activity exerted by their products [131]. Among non-coding elements, those that have been most extensively studied in HGOS are microRNAs (miRNAs) and long non-coding RNAs (lncRNAs). Although some evidence of the possible involvement of non-coding RNAs in HGOS pathogenesis has been reported [132,133,134,135], very few data have been provided so far regarding the potential impact of polymorphisms affecting miRNAs or lncRNAs on HGOS survival.

A recent study evaluated the correlation between SNPs of hsa-miR-124a and risk and prognosis of HGOS [136]. SNPs of hsa-miR-124a were assessed on 174 HGOS patients and 150 healthy people. The CG+GG genotype of the hsa-miR-124a rs531564 polymorphism proved to be associated with a decreased risk for HGOS development and a higher five-year survival rate compared to CC genotype [136].

Moreover, NGS techniques have shown that a single miRNA locus can generate multiple distinct miRNA isoforms (isomiRs) that differ in length, sequence composition or both of them. Multiple lines of evidence suggest that the profile of isomiRs is cell- and tissue-specific, and, therefore, can be used as a biomarker for many diseases, including cancers [137]. Studying isomiRs is therefore expected to highlight new important biomarkers, since it has been indicated that they play important roles in a wide range of biological processes, including promotion of apoptosis and repression of tumor progression [137].

## 6. Preclinical Models and Public Datasets 

The establishment of patient-derived tumor xenografts (PDXs or PDTXs) has become an innovative approach to overcome the frequent lack of adequate experimental models for HGOS [138,139]. Therefore, two recent studies will be discussed here. Nanni and co-workers [140] implanted fresh osteosarcoma or Ewing sarcoma tumor specimens sub-cutaneously at the level of trans-scapular brown fat of 5–11 week-old NOD Scid gamma (NSG) male mice. When the tumor reached a maximal volume of 2.5 cm^3^ mice were sacrificed. Established PDXs were also implanted in BALB/c Rag2-/-;Il2rg-/- (RGKO) mice. In the study by Sayles and co-workers [141], fresh or frozen osteosarcoma tumor samples were implanted in the sub-renal capsule of NSG mice after having dipped them into Matrigel. The tumor growth was monitored up to one year. Both studies reported the generation of PDX-derived cell lines with [141] or without [140] prior enrichment for human cells. 

In the study by Nanni and co-workers [140], the morphological characterization and expression of two biomarkers, SATB homeobox 2 (SATB2) and ABCB1, revealed by immunohistochemistry, as well as gene expression profiling by the Agilent whole human genome microarray (#G4851C) showed remarkable similarity between the patient’s tumor and PDX, which was maintained at least until the sixth in vivo generation. Genes differentially expressed in the original tumor compared to the corresponding PDXs prevalently belonged to immune functional categories, due to the gradual replacement of human leukocytes by murine leukocytes during growth in mice. PDX-derived cell lines maintained the features of the original tumor at a lower degree indicating that adaption to in vitro growth has a higher impact on the molecular profile than adaption to in vivo growth. Primary cell cultures appeared less reliable than PDXs, which might be attributed to the high genetic instability of HGOS.

In the study by Sayles and co-workers [141], WGS was performed on 30 tumor samples and corresponding germline DNAs obtained from 23 patients. This dataset was expanded by the non-overlapping dataset of 33 samples obtained from 31 patients [142] with the aim to identify recurrent copy number (CN) alterations in druggable and clinically actionable cancer genes. The most commonly amplified genes were *MYC* (39%) and cyclin E1 (*CCNE1;* 33%), followed by RAD21 cohesin complex component (*RAD21*; 38%), *VEGFA* (23%), Aurora kinase B (*AURKB*; 13%) and cyclin-dependent kinase 4 (*CDK4*; 11%). Recurrent deletions, structural variations (SV) and somatic nucleotide variants (SNV) were found most frequently in the tumor suppressor genes *TP53* (74%), *RB1* (64%) and phosphatase and tensin homolog (*PTEN*; 56%). Fifteen PDX models were sequenced, where possible, of multiple passages and data were compared with the corresponding primary tumors. These analyses revealed that CN changes in PDX were highly correlated with those found in the corresponding primary tumors. In cases with RNA-sequencing data, a positive correlation was found between somatic copy number aberrations (SCNAs) and gene expression between the PDX and their matched primary tumor. In general, genes with highly increased CNs were also overexpressed.

In both studies, PDX-bearing mice were successfully used for in vivo drug testing. Summarizing, establishment of HGOS-derived PDXs is expensive and time-consuming, thus impossible to be translated directly to the clinics. However, well characterized panels of matched tumors, PDXs and cell lines from the same patient will serve as important experimental models not only to increment our molecular knowledge of this tumor and to study its evolution, but especially for testing innovative therapeutic approaches [143,144,145]. Sayles and co-workers [141] demonstrated successfully the utility of a target genome-informed approach for the identified target genes *MYC, Cyclin-E, CDK4, AURKB* and the phosphatidylinositol 3 kinase—AKT serine/threonine kinase—mammalian target of rapamycin (*PI3K-AKT-mTOR*) and *VEGF* pathways.

To our knowledge, so far pharmacogenomic studies have generally been performed on clinical tumor specimens and not on experimental models. The same holds true for 3D cell culture or organoids. However, it is expected that all these innovative experimental models, which better mimic the clinical situation compared to classical 2D cell cultures [146,147], would maintain the patients’ pharmacogenetic genotype. Therefore, PDX-derived cell lines could eventually become a new source for pharmacogenomic-driven drug-testing in the field of translational research and hopefully applicable soon also in the clinics. 

Integrative elaboration of WGS data with multiple datasets and defined clinical parameters is an innovative approach that permits to better identify pathway associations and to provide the opportunity for discovery of clinically actionable variants and thus overcomes the limitations of gene or pathway driven studies. This approach has recently been applied to two germline datasets, Therapeutically Applicable Research to Generate Effective Treatments (TARGET) and INOVA, of patients with HGOS treated with standard methotrexate-adriamycin-cisplatin therapy [148]. Haplotype and single SNP analyses were performed, and sanger sequencing technique was used for data validation. Intronic and intergenic hotspot regions from 26 genes that were common to both data sets were significantly associated with relapse. A total of 281 variants were significantly associated with tumor necrosis. Five of these were also significantly associated with worse survival. It is noteworthy that none of these genes, *SLC22A1,* solute carrier family 22 member 8 (*SLC22A8*), UDP glucuronosyltransferase family 2 member B15 (*UGT2B15*) and carbohydrate sulfotransferase 12 (*CHST12*), has ever been studied in candidate or pathway driven approaches.

The advantage of public available datasets is that they can easily be downloaded and elaborated without the need of conducting one’s own experiments, thus avoiding high costs and technical problems. Elaborating the same data sets by different algorithms enables bioinformatics to collaborate and to improve their tools.

## 7. Social-Economical Aspects of Pharmacogenomics-Based Precision Medicine in HGOS

Most anticancer drugs, including those used in HGOS chemotherapy, have a narrow therapeutic window and exhibit variable efficacy among patients which can result in treatment failure or the development of severe toxicities. 

While the consequences of treatment failure are easily understandable, those of adverse reactions to chemotherapeutic drugs are frequently underestimated. Globally, the development of chemotherapy-related toxicities has been calculated to be responsible for about 7% of all hospital admissions in Europe and for more than 100,000 death/year in the United States [149,150]. Moreover, a large survey performed in the United States during the 1990s indicated that adverse drug toxicities ranked from fourth to sixth as leading cause of in-hospital mortality [150], and a follow-up study performed in 2010 did not show any improvement [151]. 

It has also to be taken into consideration that toxicity reactions lead to additional pharmacologic treatments to counteract their effects, which are frequently associated with a progressively worsening of patients’ quality of life and a significant increase of costs for medical care, both of which cause growing concerns about the rising expenses in oncology. 

Tailored treatments (which are included in the so-called personalized medicine or precision medicine approaches), in which biomarkers are used to match therapies to specific patient characteristics [152], may overcome at least part of these problems. Treatments modulated on the basis of individual patients’ characteristics are therefore particularly warranted to maximize the efficacy and safety of each therapeutic regimen and, consequently, to also reduce costs. The promise of pharmacogenetics and pharmacogenomics is thus in line with this scenery. 

Present drug therapies of HGOS encounter all the aforementioned problems, which are additionally enhanced by the fact that this sarcoma mainly affects young people. Like in other tumors, several scientific, social and economic aspects have to be considered to define the actual feasibility of pharmacogenetics/pharmacogenomics-based precision medicine in HGOS.

First, once predictive biomarkers have been sufficiently validated, before translating them to clinical practice it will be necessary to assess their prognostic influence in each patient subgroup and among different ethnicities in order to estimate their actual clinical value.

Another item to be considered is the cost of the biomarker test(s), which should be characterized by high sensitivity and specificity, in order to rule out or reduce as much as possible the occurrence of false-negative or false-positive results.

The costs of agents used for each biomarker(s)-driven treatment arm have also to be quantified, and programs for reimbursement of expenses for both biomarker test(s) and treatment modulation need to be established inside each country or, even, each institution.

On the other hand, it has to be considered that biomarker(s)-driven therapies will have to be considered for the small fraction of those HGOS patients, who did not sufficiently respond to conventional treatments. Therefore, for a rare tumor like HGOS, the number of patients that may be recruited inside these protocols will remain low. This fact may have both positive and negative consequences. For pharmaceutical companies producing the agents for tailored therapies, this fact can appear as poorly attractive, because of the small market and sales volume. For national-regional health systems, however, this situation may be considered as acceptable because the therapies costs will be more affordable due to the low patient numbers.

Precision medicine in HGOS may also take advantage from the development of nanomedicine and nanovectors. Since nanomedicine can modulate the biodistribution at the target site of anticancer drugs, thereby reducing their toxicity, its development will be of great help to indicate new methods of cancer treatment [153]. Nanoparticles loaded with different chemotherapeutic drugs have indeed been demonstrated to exhibit relevant anticancer activity in preclinical settings of lymphoma, glioma, and pediatric HGOS [153]. Although nanoplatforms for the delivery of chemotherapeutic drugs, small RNA molecules, photothermal therapeutics and immunotherapeutics should be further improved [153,154], the recent advances achieved in nanomedicine and nanovectors research offer the possibility to develop novel therapeutic approaches which may be considered for specific subgroups of HGOS patients stratified according their pharmacogenetic and pharmacogenomic characteristics.

## 8. Future Directions

Traditionally, pharmacogenetic and pharmacogenomic studies have focused on a few or on a small number of candidate genes, generally selected on the basis of their involvement in mechanisms related to drug action, resistance, or metabolism in either tumor or normal tissues. This approach, when applied to HGOS, has provided a series of evidence of possible clinical impact, most of which (if not all) needs, however, to be further validated.

The body of information concerning single candidate genes may further be implemented by defining genetic risk scores, which consider multiple genetic variants that individually contribute to a specific phenotype and may increase their clinical impact when considered together. 

The future translation to HGOS clinical practice of pharmacogenetic and pharmacogenomic provided evidence needs to proceed along some necessary steps. 

First, ongoing clinical trials should incorporate pharmacogenetic and pharmacogenomic analyses in order to validate the findings reported in single, frequently not large, studies or emerged from preclinical experimental models.

Only once a marker or a group of markers will be validated, it may be proposed for a genetically-guided decision of personalized therapies aimed to enhance treatment success rate and simultaneously decrease the frequency of collateral toxicities, with a consequent improvement of patients’ quality of life and a reduction of health care costs.

### 8.1. Genetic Testing and Clinical Trials

Genetic testing of patient-derived tumor cells can be used to identify specific therapeutic targets and, consequently, to identify patients who may benefit from treatments targeting their specific tumor genetic alterations. By using this approach, several MATCH clinical trials (targeted therapy directed by genetic testing in treating pediatric patients with relapsed or refractory advanced solid tumors, non-Hodgkin lymphomas, or histiocytic disorders; ClinicalTrials.gov Identifier: NCT03155620) have been launched and are presently active and ongoing in HGOS (Table 2). The pediatric MATCH screening trials are a complex of phase II clinical studies, in which treatment is directed by genetic testing. These trials are recruiting pediatric patients with solid tumors (including HGOS), non-Hodgkin lymphomas, or histiocytic disorders, who have progressed following at least one line of standard systemic therapy and/or for whom no standard rescue treatments are available. 

Similarly, NGS technologies applied to pharmacogenetics and pharmacogenomics are also expected to provide information that can be translated to clinical care. Some experience in improving drug safety and efficacy by means of the so-called genomic medicine has been recently described [155].

NGS techniques are expected to generate new findings and indicate genetic alterations, which may be of help to better delineate the genes and biochemical pathways important for HGOS growth, progression, clinical behavior and, most of all, biomarkers that may predict which therapy will have the highest probability to obtain the major benefits with minimal risk of toxicity in genetically-stratified subgroups of patients.

Moreover, these methodological approaches may indicate new potential therapeutic targets, which could be taken into consideration to plan novel tailored treatment strategies aimed to improve outcome of patients with localized HGOS and, even more, of those with metastatic disease, for whom really effective therapeutic options are still limited.

Part of the aforementioned information is expected to derive from the Therapeutically Applicable Research to Generate Effective Treatments (TARGET) program (http://ocg.cancer.gov/programs/target/projects/osteosarcoma; ClinicalTrials.gov Identifier: NCT01190943), a multicenter collaborative project aimed to the identification of genetic and epigenetic aberrations in HGOS through a combination of different genomic approaches. The objective of this study is to identify those aberrations that are involved in the pathogenesis of HGOS and which may play a role in chemoresistance and metastasis using high-resolution, genome-wide technologies. The final aim of the study is to indicate biomarkers to be validated as new therapeutic targets for patients with HGOS, especially for those with metastatic disease and/or unresponsive to standard chemotherapy.

The Individualized Therapy for Relapsed Malignancies in Childhood (INFORM) program (https://www.kitz-heidelberg.de/en/for-physicians/clinical-studies/molecular-diagnostics-studies/inform/) [156] is another multicentric study that aims to identify novel therapeutic targets and to use them to drive clinical management of patients with pediatric malignant tumors, including HGOS [157].

The UK 100,000 Genomes Cancer Project (https://www.genomicsengland.co.uk/about-genomics-england/the-100000-genomes-project/) [158] also plans to bring whole genome sequencing-provided evidence directly into clinical care in several rare diseases, as well as in advanced solid tumors and hematological malignancies [159].

The Geisinger MyCode Community Health Initiative (https://www.geisinger.org/mycode) [160] is a precision medicine project which is ongoing at Geisinger locations in Pennsylvania and New Jersey. One of the major objectives of this program is the establishment of a system-wide biobank designed to store blood and other samples from a large number of patients and participants (more than 190,000 patients/participants have already agreed and signed up to join this study). The final aim of this program is the use of DNA genomic analyses to improve healthcare through early diagnosis of different medical conditions and the indication of new treatments to manage each disease [161,162,163]. 

Very recently, the results of the Sarcoma Decision Impact Clinical Trial study have been published by the Foundation Medicine group [164]. This study applied the strategy of comprehensive genomic profiling to sequence both DNA and RNA from sarcoma tumor samples, with the aim to identify known and novel alterations that may drive oncogenicity and potentially impact on physicians’ treatment decision-making. The authors assessed the feasibility of this approach in a larger group of 392 bone and soft-tissue sarcoma patients (including 16 patients with HGOS), showing that the success rate for bone tumor samples was 65.3%. 

In a subgroup of 28 patients with different malignancies, the impact of genomic profiling data on physicians’ treatment decision-making was also assessed. Physicians decided to switch treatment on the basis of comprehensive genomic profiling in seven cases (25%). 

The first conclusion addressed by this study was that comprehensive genomic profiling can be applied to bone tumors, for which, however, careful sample acquisition with attention to decalcification and nucleic acid handling are mandatory to ensure usable results. The second main indication provided by this report was that comprehensive genomic profiling can impact on physician decision-making regarding treatment, in particular in relapsed patients.

### 8.2. Epigenetics 

Epigenetic studies are also expected to provide information of possible clinical value in the near future. Research on non-coding RNAs in HGOS has been rapidly expanding and indicating miRNAs and lncRNAs, which might be considered as promising future candidate therapeutic targets [133,134,165]. Studies on polymorphisms of non-coding RNAs in HGOS are still at their beginning, but they are sure to increase in the next years. However, there are at least two major items that must be taken into consideration for a possible clinical translation of the provided evidence:

1. It is mandatory to determine and validate the actual value of miRNAs and lncRNAs that have been highlighted in HGOS experimental models and clinical samples as possible therapeutic targets;
2. The design of non-coding RNA-based or targeted treatments with sufficient efficiency, delivery, therapeutic effects, and safety profiles to be transferred to the clinic is mostly still at a preclinical phase of development.

Targeting non-coding RNAs can be performed by using small interfering RNAs, antisense oligonucleotides, ribozymes, or aptamers, as well as by using clustered regularly interspaced palindromic repeats (CRISPR)/CRISPR-associated protein 9 (Cas9) methodologies [133,165,166]. Research in this field has been rapidly expanding and it will further enhance in the coming years. Therefore, there is hope that at least few of these approaches will be sufficiently developed and validated to be safely translated to the clinic in a medium-term period.

### 8.3. Immunopharmacogenomics

In addition to the aforementioned applications, NGS technologies have also been applied to the genetic characterization of the immune system inside a new field of research known as immunogenomics or immunopharmacogenomics [167]. Since host immune responses has been recognized to be important not only for immunotherapy efficacy, but also as mediators for cytotoxic agents and targeted drugs activity, immunopharmacogenomics will hopefully yield useful information to monitor tumor treatment response and predict clinical outcome. In addition, this approach may contribute to the identification of tumor neoantigens that may be proposed as novel immunotherapeutic targets.

By using NGS, it will also be possible to estimate the tumor mutational burden (TMB), which is an index of the number of mutations in a specific tumor genome. This possibility has a relevant clinical impact because it has been observed that higher numbers of somatic mutations are correlated with a better clinical response to monoclonal antibodies which block immune checkpoint molecules such as the CTLA-4, programmed cell death-1 (PD-1), and programmed death-ligand 1 (PD-L1) [168,169,170]. 

Despite the initial expectancies, immunotherapy has unfortunately not yet provided significant improvements in HGOS cure rate. These unsatisfactory results can be explained by the low TMB exhibited by the majority of HGOS patients [171,172]. Determining TMB with NGS, an approach which is already feasible in many laboratories and institutes, may therefore be of great help to identify those HGOS patients with a higher probability to respond to immunotherapy and drive the choice of the most appropriate immunotherapeutic approach on the basis of patients’ genomic characteristics.

### 8.4. Paired Sequencing

A strategy that may speed up the identification of tumor-related biomarkers can also consist in paired sequencing of blood and tumor cells from the same patient, enabling the identification of the mutations and genetic alterations that are specifically acquired in the malignant cells but also those present at germline level.

Moreover, pharmacogenetic and pharmacogenomic information obtained from paired normal and tumor samples of the same patient will allow researchers to establish a direct relationship between the tumor genotype and a drug’s efficacy, or between the constitutive genotype and the development of toxicity reactions. This body of evidence will be essential to modulate and optimize chemotherapy through the identification of the most responsive patients and those who have higher probabilities to develop treatment-related toxicities.

A fact that needs to be taken into consideration is that, since DNA is easily obtainable via a blood sample, saliva or buccal swab, the analysis of germline polymorphisms can routinely be applied to patients also during chemotherapy treatment, providing a predictive tool which may decrease the occurrence of adverse toxicities and increase drug efficacy and safety.

## 9. Conclusions

The number of mutations or genetic alterations that can be considered as tumor drivers has significantly increased in the last decade thanks to the development of new techniques, as well as through the data generated by large international sequencing studies, such as the Cancer Genome Atlas [173] or the International Cancer Genome Consortium projects [174]. 

Several efforts are thus ongoing in HGOS preclinical and clinical research in order to identify genetic changes that can be used as biomarkers to predict tumor behavior, prognosis, drug response and toxicity development, which may enable early monitoring for recurrence of disease and, finally, be indicated as new candidate therapeutic targets. From this point of view, genome-wide technologies may be of great help for extracting genetic signatures that can be used as predictive biomarkers and identify patients eligible for specific targeted treatments [17].

Economical aspects have also to be considered. It is evident that pharmacogenetic and pharmacogenomic profiling has the potential to improve the treatment results in human tumors, as well as in other diseases. In this respect, the methods used in these studies must, however, be considered in cost-effectiveness evaluations of treatment driven by pharmacogenetic and pharmacogenomic biomarkers. The progressive decrease of the costs of the new technologies for genomic analysis is a positive trend that gives hope for the adoption of pharmacogenetic and pharmacogenomic profiling in clinical practice in the short-to-medium term. 

## Figures and Tables

**Table 1:** Rare cancer predisposition syndromes associated with osteosarcoma.

Autosomal Dominant (*Gene Involved*)	Incidence of HGOS	Estimated Fold-Risk	Autosomal Recessive (*Gene Involved*)	Incidence of HGOS
Li-Fraumeni Syndrome (*TP53*)	3% [17] 5–11% [18]	107 [19]	Rothmund-Thomson Syndrome (*RECQL4*)	32% [20] 48% [21]
Hereditary Retinoblastoma (*RB1*)	12% [17]	200–400* [22] 69–400* [19]	Rapadilino Syndrome (*RECQL4*)	13% [22] 40% [21]
Diamond-Blackfan anemia (genes encoding ribosomal proteins, of which the most common is *RPS19*, and *GATA1*)	<1% [23] 14% [24]	42 [24]	Bloom Syndrome (*RECQL3*)	<12% [25]
			Werner Syndrome (*RECQL2*)	7% [22]

**Footnote:** estimated fold-risk values were available for autosomal dominant syndromes only. **Legend:** *after radiation therapy. **Abbreviations:** HGOS, high-grade osteosarcoma; *TP53*, tumor protein 53; *RB1*, retinoblastoma transcriptional corepressor 1; *RPS19*, ribosomal protein S19; *GATA1*, GATA binding protein 1; *RECQL*, RecQ like helicase.

**Table 2:** List of the pediatric MATCH screening trials that are presently active and recruiting patients with relapsed or refractory advanced solid tumors, including high-grade osteosarcoma. All trials are carried out in the USA. Time period refers to the actual study start date and estimated study completion date.

ClinicalTrials.Gov NCT Identifier	Drug	Mechanism of Drug Action and Trial Description	Stage of Development (Time Period)
NCT03210714	**Erdafitinib** (JNJ-42756493)	Inhibition of *FGFR* with negative effects on tumor cell growth and angiogenesis. This trial studies the activity of erdafitinib in patients with *FGFR* gene mutations, who are affected by disseminated tumors.	Phase II (11/2017–12/2024)
NCT03213665	**Tazemetostat** (EPZ-6438)	Inhibition of the activity of human polycomb repressive complex 2 containing wild-type histone-lysine N-methyltransferases EZH1 and EZH2, with a consequent inhibition of tumor cells growth. This trial is intended to assess the activity of tazemetostat in patients with *EZH2*, *SMARCB1*, or *SMARCA4* gene mutations, who are affected by disseminated tumors.	Phase II (07/2017–09/2024)
NCT03213678	**Samotolisib** (LY3023414)	Inhibition of PI3K/AKT/mTOR pathway, producing negative effects on the growth of cancer cells. This trial evaluates the activity of samotolisib in patients with *TSC* or *PI3K/mTOR* mutations, who developed metastasis or local recurrences, or who are refractory to conventional treatments.	Phase II (07/2017–09/2024)
NCT03220035	**Vemurafenib** (PLX40321; trade name Zelboraf^®^)	Inhibition of the mutated B-Raf protein, interrupting its stimulation of cell growth. This trial evaluates how vemurafenib works in treating patients with *BRAF V600* mutations who are affected by tumors that have disseminated in the body or do not respond to conventional treatments.	Phase II (07/2017–12/2023)
NCT03233204	**Olaparib** (AZD-2281, MK-7339, trade name Lynparza^®^)	Inhibition of PARP1 and consequent impairment of DNA repair activity. This trial assesses the efficacy of olaparib in treating patients with hereditary *BRCA1* or *BRCA2* mutations, leading to defects in DNA damage repair activity. The trial recruits patients who have developed metastasis or local recurrences, or who are refractory to conventional treatments.	Phase II (07/2017–09/2024)
NCT03526250	**Palbociclib** (PD-0332991, trade name Ibrance^®^)	Selective inhibition of the cyclin-dependent kinases CDK4 and CDK6. By inhibiting CDK4 and CDK6, palbociclib may stop the growth of cancer cells. This trial evaluates how palbociclib works in treating patients with *RB1*-positive tumors which have disseminated in the body or do not respond to conventional treatments.	Phase II (06/2018–06/2025)
NCT03698994	**Ulixertinib** (BVD-523; VRT752271)	Inhibition of ERK1/2 kinases, belonging to the MAPK pathway. By inhibiting cancer cells harboring mutations in the MAPK pathway, ulixertinib may stop tumor growth. This trial is intended to estimate the activity of ulixertinib in treating patients with disseminated tumors, who present genetic alterations in the MAPK signaling pathway.	Phase II (10/2018–12/2025)

*AKT*, AKT serine/threonine kinase; *BRCA*, breast related cancer antigen; *CDK*, cyclin-dependent kinase; *ERK*, extracellular signal-regulated kinase; *EZH1*, enhancer of zeste 1 polycomb repressive complex 2 subunit; *EZH2*, enhancer of zeste 2 polycomb repressive complex 2 subunit; *FGFR*, fibroblast growth factor receptor; *mTOR*, mammalian target of rapamycin; MAPK, mitogen-activated protein kinase; *PARP1*, poly (ADP-ribose) polymerase 1; *PI3K*, phosphatidylinositol 3-kinase; *SMARCB1*, SWI/SNF related matrix associated actin dependent regulator of chromatin, subfamily B, member 1; *SMARCA4*, SWI/SNF related matrix associated actin dependent regulator of chromatin, subfamily A, member 4; *B-Raf*, B-Raf proto-oncogene, serine/threonine kinase; *RB1*, RB transcriptional corepressor 1 (also known as retinoblastoma gene 1); *TSC*, TSC complex subunit.

## Abbreviations

ABC	ATP-binding cassette
ABCB1	ATP-binding cassette, subfamily B (MDR/TAP), member 1
ABCC2	ATP-binding cassette, subfamily C (CFTR/MRP), member 2
ABCC3	ATP-binding cassette, subfamily C (CFTR/MRP), member 3
ABCC5	ATP-binding cassette, subfamily C (CFTR/MRP), member 5
ACT	anthracycline-induced cardiotoxicity
ACYP2	acylphosphatase 2
ADME	absorption, distribution, metabolism, excretion
AKT	AKT serine/threonine kinase
ALT	alanine transaminase
ATM	ataxia telangiectasia mutated)
ATR	ataxia telangiectasia and Rad3 related
ATRX	ATRX chromatin remodeler
AURKB	Aurora kinase B
B-Raf	B-Raf proto-oncogene, serine/threonine kinase
BRCA1	breast related cancer antigen 1
BRCA 2	breast related cancer antigen 2
Cas9	CRISPR associated protein 9
CBRs	carbonyl reductases
CCNE1	cyclin E1
CDK4	cyclin-dependent kinase 4
CDK6	cyclin-dependent kinase 6
CDKN2A	cyclin dependent kinase inhibitor 2A
CHST12	carbohydrate sulfotransferase 12
CI	confidence interval
CN	copy number
COMT	catechol O-methyltransferase
COSMIC	catalogue of somatic mutations in cancer
COX8A	cytochrome C oxidase subunit 8A
CRISPR	clustered regularly interspaced palindromic repeats
CTLA-4	cytotoxic T-lymphocyte antigen 4
CYP	cytochrome P450
DHFR	dihydrofolate reductase
DMEs	drug metabolizing enzymes
ERCC 1	excision repair cross-complementation group 1
ERCC 2	excision repair cross-complementation group 2
ERCC 5	excision repair cross-complementation group 5
ERK	extracellular signal-regulated kinase
EZH1	enhancer of zeste 1 polycomb repressive complex 2 subunit
EZH2	enhancer of zeste 2 polycomb repressive complex 2 subunit
FGFR	fibroblast growth factor receptor
GATA1	GATA binding protein 1
GGH	gamma-glutamyl hydrolase
GRM4	glutamate metabotropic receptor 4
GST	glutathione S-transferase
GSTM1	glutathione S-transferase M1
GSTM3	glutathione S-transferase M3
GSTP1	glutathione S-transferase P1
GSTT1	glutathione S-transferase T1
GWAS	genome-wide association study
HAS3	hyaluron synthase 3
HGOS	high-grade osteosarcoma
IL-8	interleukin-8
INFORM	INdividualized therapy FOr Relapsed Malignancies
lncRNAs	long non-coding RNAs
LP	likely pathogenic
LRP2	LDL receptor related protein 2, or megalin
MAPK	mitogen-activated protein kinase
MDM2	mouse double minute 2 (also known as murine double minute 2)
MDM4	mouse double minute 4
MEN1	menin 1
miRNAs	microRNAs
MMS19	nucleotide excision repair homolog
MSH2	mutS homolog 2
MTHFD1	methylenetetrahydrofolate dehydrogenase (NADP+ dependent) 1
MTHFR	methylenetetrahydrofolate reductase
mTOR	mammalian target of rapamycin (mechanistic target of rapamycin)
MTX	methotrexate
NBN	nibrin
NER	nucleotide excision repair
NF1	neurofibromin 1
NFE	non-Finish European
NGS	next generation sequencing
NSG	NOD Scid gamma
odd ratio	OR
P	pathogenic
PARP1	poly (ADP-ribose) polymerase 1
PD-1	programmed cell death-1
PD-L1	programmed death-ligand 1
PDXs or PDTXs	patient-derived tumor xenografts
PI3K	phosphatidylinositol 3 kinase
PMS2	PMS1 homolog 2, mismatch repair system component
POT1	protection of telomeres 1
PRCKG	protein kinase CGMP-dependent 1
PTEN	phosphatase and tensin homolog
RAD21	RAD21 cohesin complex component
RARG	retinoic acid receptorγ
RB1	RB transcriptional corepressor 1 (also known as retinoblastoma gene 1)
RECQL2	RecQ like helicase 2
RECQL3	RecQ like helicase 3
RECQL4	RecQ like helicase 4
RECQL5	RecQ like helicase 5
RFC	reduced folate carrier
RPS19	ribosomal protein S19
RPS26	ribosomal protein S26
RUNX1	RUNX family transcription factor 1
SATB2	SATB homeobox 2
SCNAs	somatic copy number aberrations
SLC19A1	solute carrier family 19 (folate transporter) member 1 (also known as reduced folate carrier, RFC or reduced folate carrier 1, RFC1)
SLC22A17	solute carrier family 22 member 17
SLC22A2	solute carrier family 22 member 2
SLC22A7	solute carrier family 22 member 7
SLC22A8	solute carrier family 22 member 8
SLC28A3	solute carrier family 28 member 3
SLCO1B1	solute carrier organic anion transporter family member 1B1
SMARCA4	SWI/SNF related matrix associated actin dependent regulator of chromatin, subfamily A, member 4
SMARCB1	SWI/SNF related matrix associated actin dependent regulator of chromatin, subfamily B, member 1
SNPs	single nucleotide polymorphisms
SNV	somatic nucleotide variants
SV	structural variations
TARGET	Therapeutically Applicable Research to Generate Effective Treatments
TGFB1	transforming growth factor beta 1
TMB	tumor mutational burden
TNF	tumor necrosis factor
TP53	tumor protein 53
TPMT	thiopurine S-methyltransferase
TS	thymidylate synthase
TSC	TSC complex subunit
UGT1A6	UDP glucuronosyltransferase 1 A6
UGT2B15	UDP glucuronosyltransferase family 2 member B15
VEGF	vascular endothelial growth factor
VHL	Von Hippel-Lindau tumor suppressor
WES	whole-exome sequencing
WGS	whole-genome sequencing
XPC	xeroderma pigmentosum complementation group C
XRCC3	X-ray repair cross complementing 3
